# The Role of Concussion History and Biological Sex on Pupillary Light Reflex Metrics in Adolescent Rugby Players: A Cross-Sectional Study

**DOI:** 10.3390/sports12020056

**Published:** 2024-02-11

**Authors:** Connor McKee, Mark Matthews, Alan Rankin, Chris Bleakley

**Affiliations:** 1 Faculty of Life and Health Sciences, Ulster University, Belfast BT15 1ED, UK; mckee-c46@ulster.ac.uk (C.M.); m.matthews@ulster.ac.uk (M.M.); 2 Sports Institute of Northern Ireland, Jordanstown BT9 5LA, UK; alanrankin@doctors.org.uk; 3 Sport Medicine NI Ltd., Belfast BT6 9HL, UK

**Keywords:** recovery, female, oculomotor, pupillometer, objective, athlete, sport, digital

## Abstract

**Background:** Concussion examination is based primarily on clinical evaluation and symptomatic reporting. Pupillary light reflex (PLR) metrics may provide an objective physiological marker to inform concussion diagnosis and recovery, but few studies have assessed PLR, and normative data are lacking, particularly for adolescents. **Aim:** To capture PLR data in adolescent rugby players and examine the effects of concussion history and biological sex. **Design:** Cross-sectional. **Methods:** Male and female adolescent rugby union players aged 16 to 18 years were recruited at the start of the 2022–2023 playing season. PLR was recorded using a handheld pupillometer which provided seven different metrics relating to pupil diameter, constriction/dilation latency, and velocity. Data were analysed using a series of 2 × 2 ANOVAs to examine the main effects of independent variables: biological sex, concussion history, and their interactions, using adjusted *p*-values (*p* < 0.05). **Results:** 149 participants (75% male) were included. A total of 42% reported at least one previous concussion. Most metrics were unaffected by the independent variables. There were however significant main effects for concussion history (F = 4.11 (1); *p* = 0.05) and sex (F = 5.42 (1); *p* = 0.02) in end pupil diameters, and a main effect for sex in initial pupil diameters (F = 4.45 (1); *p* = 0.04). Although no significant interaction effects were found, on average, females with a concussion history presented with greater pupillary diameters and velocity metrics, with many pairwise comparisons showing large effects (SMD > 0.8). **Conclusions:** Pupillary diameters in adolescent athletes were significantly affected by concussion history and sex. The most extreme PLR metrics were recorded in females with a history of concussion (higher pupillary diameters and velocities). This highlights the importance of establishing baseline PLR metrics prior to interpretation of the PLR post-concussion. Long-standing PLR abnormalities post-concussion may reflect ongoing autonomic nervous system dysfunction. This warrants further investigation in longitudinal studies.

## 1. Introduction

Sport-related concussion is defined as a complex pathophysiological process affecting the brain, induced by traumatic biomechanical forces [1,2]. Many concussions occur during contact and collision sports such as American football, ice hockey, and rugby union [3]. The rate of concussion in professional rugby union is estimated at 21.5 per 1000 game hours [4]. The incidence of concussion is also high in adolescent rugby and prospective research has reported 6.1 concussions per 1000 game hours, which is equivalent to around 3 concussions per team per season [5]. While clinical recovery after sport-related concussion in adolescents is commonly reported to occur within 30 days of a concussion [2], there is a subgroup of around 30% of people who continue to suffer persistent post-concussion symptoms [6]. 

Concussion can affect multiple systems, including vestibular, cognitive, and oculomotor systems [7,8]. This can result in headaches, dizziness, balance problems, and eye disturbances [2,9], such as blurry or double vision and sensitivity to light. Dysfunction of the autonomic nervous system (ANS) has been identified in individuals post-sport-related concussion [10]. The ANS regulates many body organ systems including blood pressure, gastrointestinal movement, body temperature, metabolism, glucose control, and cerebral blood flow [11]. ANS function can be assessed using various biomarkers, including heart rate variability, arterial pulse wave, or by monitoring an individual’s vascular response (blood pressure, heart rate) to changes in posture (head-upright tilt-table testing) or other physiological stressors such as the Valsalva manoeuvre [12]. The pupillary light reflex (PLR) test examines the pupil’s reaction to a brief light exposure, and is one of the easiest methods to assess ANS function [13]. PLR plays a significant role in eye convergence and accommodation and is regulated by both the sympathetic and parasympathetic systems. PLR was traditionally assessed subjectively using a pen light test, but infrared pupillometry now provides a more objective and reliable quantification of the pupillary response [14].

Current evidence suggests that PLR metrics are affected by concussions. In a 2017 review of controlled trials, Ciuffreda et al. [15] concluded that concussion is associated with delayed responsivity and smaller baseline diameter, compared with normal controls. This is corroborated in more recent research. A case-controlled study involving military personnel found that pupillary response markers were markedly affected by acute mTBI, with the largest effects observed in constriction and dilation velocities and in 75% re-dilation times [16]. Similar patterns were reported in a large retrospective review of clinical cases where the author also noted differences in the maximum and minimum pupil diameters between subjects who had suffered a concussion and those who had not, with most persisting over the life span [17]. 

Studies examining the effects of concussion on the PLR have largely involved male adults but PLR metrics are also modulated by age and sex [14,18,19]. Some data suggest PLR metrics are largely stable across infancy [18], whereas others have reported changes occurring at the time of adolescence. In one study, male adolescents aged 12 to 18 years had larger maximum pupil diameters, slower maximum constriction velocities, and smaller percentage constriction compared to children aged 6 to 11 years [18]. The effect of biological sex on the PLR is not clear: initial studies recorded larger pupil diameters [17,20] and slower dilation velocities [20] in females compared to males, whereas results have been mixed for other key metrics such as time to 75% re-dilation [20,21].

Existing evidence supports the use of the PLR as a rapid, objective physiological indicator for sport-related concussions, which can inform diagnostic and management decisions. However, baseline differences must be considered when interpreting post-concussion biomarkers and symptoms. There is therefore a need to establish normative baseline PLR values for adolescents and quantify the effects of key moderating variables. The aim of this study is to capture baseline PLR data (using a handheld pupillometer) in a sample of adolescent rugby players, and to examine if outcome metrics are affected by concussion history, biological sex, and their interaction. 

## 2. Methods

This was a cross-sectional cohort study undertaken over a single rugby playing season (2022–2023). Male and female participants were recruited from 9 school and club rugby teams across the province of Ulster in Northern Ireland. To be eligible participants were required to be playing at First XV level or equivalent (aged 16–18 years). Ethical approval for this research was granted by the Ulster University Research Ethics Committee. This research is part of a larger longitudinal study that is tracking recovery post-concussion, and the protocol has been published previously [22].

### 2.1. Participants

Participants were recruited from school and club rugby teams across the Province of Ulster. There are more than 30 school teams with male adolescent rugby players, and 25 registered clubs with dedicated female youth rugby teams, registered across the province of Ulster. Many of these sites have previously been active members of the Rugby Injury Surveillance in Ulster Schools (RISUS) and have successfully collaborated in several previous research studies [5,9,23]. We recruited a convenience sample from this sampling frame. Of the 15 rugby union teams and clubs initially contacted, 10 (67%) agreed to participate. A total of 149 participants (n = 113 male (75.8%), n = 36 female (24.2%)) aged 16 to 18 years (M = 17.2 ± 0.7) from the 10 teams/clubs were eligible. Inclusion criteria for participation were: (i) aged between 16 and 18 years; (ii) free from injury at the time of recruitment; and (iii) currently playing rugby union in the 2022/23 playing season at a school or club in Ulster. Participants who had a concussion within the previous month or had recent eye trauma were excluded. Consenting participants completed a baseline questionnaire covering demographic characteristics (date of birth, sex, weight, height, and concussion history).

### 2.2. Data Collection

#### 2.2.1. Demographics/Medical History

Baseline testing was undertaken before the start of the 2022–2023 rugby playing season. Initially, this involved the completion of a questionnaire detailing characteristics (age, weight, and height), injury history (previous concussion (yes/no)), and time loss from sport (days) associated with a previous concussion. All definitions and procedures used were compliant with the international consensus statement on injury surveillance studies for rugby [24]. The primary researcher was present while participants completed the questionnaire and was able to answer their questions. 

#### 2.2.2. Pupillary Light Reflex Metrics

**Figure 1:**
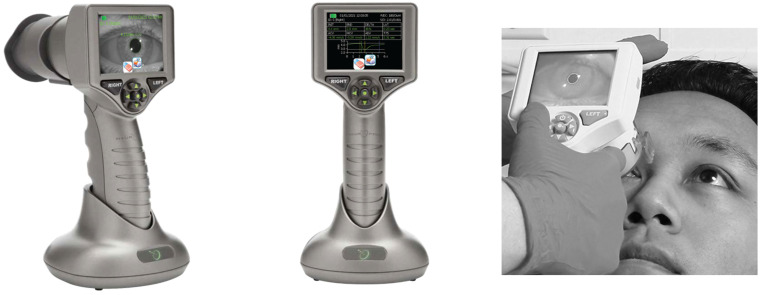
PLR device and data display.

PLR (Figure 1) was measured using a Neuroptics PLR-3000 handheld, infrared, automated, monocular pupillometer (NeurOptics, 9223 Research Drive, Irvine, CA 92618, USA), as seen in the images above. This device is approved by the United States Food and Drug Administration (FDA) and has been used in similar studies of mTBI in adults [25]. It is placed over the eye (as seen in the right picture) and emits a brief, step-input, white light stimulus (154 milliseconds’ duration; 180 microwatts’ power) and the pupillometer captures dynamic responses 32 times per second, analysing a continuous, 5 s, digital video of the pupillary response to light. The PLR device software (NeurOptics, 9223 Research Drive, Irvine, CA 92618, USA)captures 7 metrics, which are displayed instantly following the assessment on screen (as seen in the middle picture), which can also be seen in full size in Table 1. 

PLR assessments were conducted in an environment with a room illumination of approximately 350 lux (moderate photopic viewing conditions), as per previous studies [21]. Blinding was not implemented regarding the concussion status or biological sex of athletes during pupillometry assessments, as this method involves an objective measure that does not necessitate a subjective interpretation of results. Throughout testing, participants were instructed to focus on a 3 m distance target with the non-tested eye for ocular fixation and accommodation during a 5 s measurement period. Monocular measurements were repeated 3 times for each eye, alternating 1 min time intervals to allow rapid visual light adaptation, to obtain 2 to 3 artifact-free responses per eye. The combined mean of each pupillometry metric was then calculated for at least 2 assessments without artifacts, defined as blinks or eye movements occurring within the first 3 s of the response. These testing conditions were consistent with a previously published study examining pupillary light reflex metrics [21]. Athletes had come from normal school conditions prior to the collection of their pupillary light reflex metrics and had not been involved in strenuous or any form of exercise prior to the collection of data.

### 2.3. Statistical Analysis

Demographic and injury history data were summarized using means and standard deviations for scale variables and frequencies and percentages for nominal variables. Data were analysed using the Statistical Package of Social Sciences (IBM SPSS Statistics Version 29). Initially, outcome data were examined using a residuals plot (residuals vs. predicted values) to ensure that the assumptions of the homogeneity of variances and normality of residuals were met. To address our research question of how concussion history and biological sex affect PLR metrics at baseline, we used a 2 × 2 ANOVA. The independent factors were concussion history (yes, no) and biological sex (male, female). We were testing the following Null Hypothesis (H0): there is no significant interaction between “concussion history” and “gender”, and there are no main effects of either “concussion history” or “gender” on the dependent variable. For each PLR outcome, we presented the sum of squares, degrees of freedom, mean squares, and F-statistics for each of the factors (concussion history and gender) and their interaction. A significance level of 0.05, was used as a threshold for rejecting the null hypothesis. If the omnibus ANOVA revealed significant effects, we used post hoc tests (with Bonferroni corrections) to identify specific differences between group means. We also presented between-group comparisons (concussion history vs. no concussion history and male vs. female) using effect sizes based on standardised mean differences (SMD) and 95% CIs; Hedges’s g = (x1 − x2)/√((n1 − 1) × s12 + (n2 − 1) × s22)/(n1 + n2 − 2).

## 3. Results

We recruited a total of 149 participants from a convenience sample (male n = 113) with a mean age of 17.2 yrs (±0.71). Males were taller and heavier (180.58 cm ± 6.40, 83.04 kg ± 15.51) when compared with females (164.42 cm ± 6.54, 64.7 kg ± 6.73). A total of 40.9% (61/149; male n = 54, female n = 7) of all participants reported a history of at least one concussion. The average time since their last reported concussion was 18.7 months (±17.4, range 1–72 months). 

Table 2 summarises PLR metrics (means, SD) split by concussion history and biological sex.

**Figure 2:**
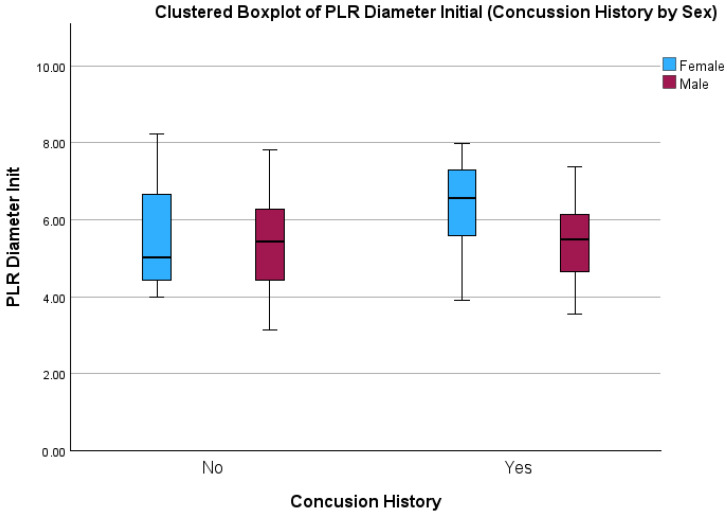
Boxplot showing Initial Pupil Diameter between males and females with and without a history of concussion.

For initial pupil diameters, there were main effects for biological sex (F = 4.45 (1); *p* = 0.04), but no main effect of concussion history (F = 2.71 (1); *p* = 0.11), or any significant interactions (F = 1.80 (1) *p* = 0.17) (Figure 2). Females with a history of concussion had the largest initial pupil diameters, with large effects size evident, when compared to males with a concussion history (SMD 0.82; 95% Cis 0.10 to 1.54), and to males (SMD 0.83; 95% Cis 0.12 to 1.55) and females with no concussion history (SMD 0.55; 95% Cis −0.22 to 1.31). 

**Figure 3:**
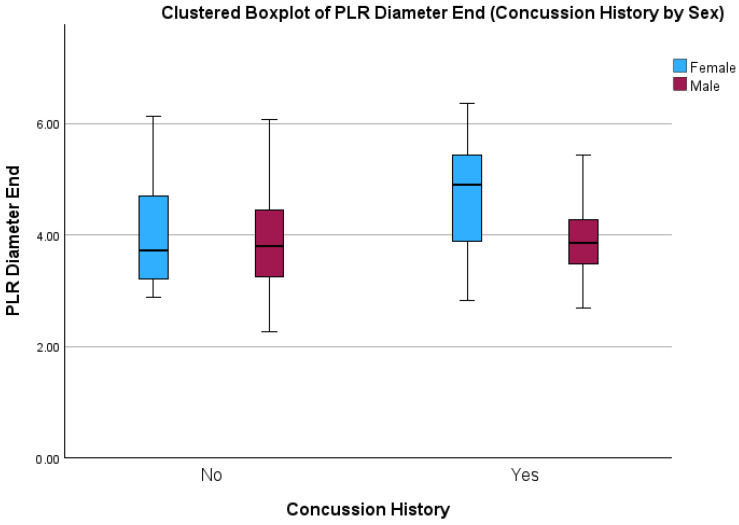
Boxplot showing End Pupil Diameter between males and females with and without a history of concussion.

There were main effects for concussion history (F = 4.11 (1); *p* = 0.05) and sex (F = 5.42 (1); *p* = 0.02) on end pupil diameter (Figure 3). On average, adolescents with a history of concussion presented with greater end pupil diameters compared to those with no history. Similarly, females had greater end pupil diameters than males. There was no significant interaction effect (concussion history x sex), but again, females with a history of concussion had the largest end pupil diameters, with large effects evident when compared to males with a concussion history (SMD 0.99; 95% CIs 0.27 to 1.7), and to males (SMD 0.74; 95% CIs 0.03 to 1.46) and females with no concussion history (SMD 0.69; 95% CIs −0.08 to 1.46). 

There were no significant main effects or interactions reported for the remaining PLR metrics (latency, average constriction velocity, maximum constriction velocity, and average dilation velocity or time to 75% re-dilation). As shown in Table 2, the constriction and dilation velocities were generally highest in females with a history of concussion, with small-to-moderate effects compared to males with a concussion history. Of note, the average time for pupil re-dilation (from minimum diameter to 75% maximum diameter) was longest in females with a concussion history, with large effects compared to males with a history of concussion (SMD 0.85; 95% CI 0.13 to 1.57).

## 4. Discussion

This is one of the first studies to capture PLR metrics via a quantitative pupillometer in adolescent rugby players compared by concussion history and biological sex. In our sample, 42% reported a history of concussion, which aligns with our previous epidemiology studies [5,23] and others examining PLR metrics in concussed adolescents [21]. We found that some PLR metrics in adolescent athletes were significantly affected by concussion history and sex. The most extreme PLR metrics were recorded in females with a history of concussion (greater pupillary diameters and higher velocities).

Concussion diagnosis is based largely on subjective clinical outcomes and a non-specific combination of symptoms [1,2]. Having access to more objective physical examinations would facilitate diagnosis [26], and may provide valid and reliable biomarkers of concussion and its sequelae. Research examining the utility of pupillary light reflex metrics as a physiologic biomarker for concussion is growing [21]. Although normative baseline data for adolescents is available for many concussion-related symptoms [18,25,27,28], there is a paucity of PLR metrics in adolescents, especially in females [20]. A small number of studies have recorded the PLR in groups of adolescents. Although we found little consistency in their demographics (athletes, non-athletes, and military cadets), equipment, testing procedure, and concussion mechanism [17,20,21,29], our average latency values were similar [17,20,21]. Although our average pupillary diameters (both initial and end) were comparable to Ishikawa [29], they were larger than in other studies [17,20,21]. Constriction velocities seem to be inconsistent across the available literature; our values exceeded some reports [20,21], but our MCV results are comparable to Carrick [17]. These differences may be due to varying photopic viewing conditions in these studies at the time of testing; some were not controlled for at all [20], with others using ambient environmental lighting situations [17], a 3 min dark-room adaptation [29], or moderate photopic viewing conditions (approximately 350 lux) [21]. We must also be cognisant that pupillary light reflex can be influenced by many other factors, including the time since concussion, various medications, or other neurological conditions.

A key finding was that adolescent rugby players with a history of concussion had a greater end pupil diameter compared to those without a history of concussion. This has been reported in other studies whereby athletes 12 days post-concussion had larger end pupillary diameters than those with no concussion [21]. Although athletes’ average time since concussion was 18.7 months, it is possible that our observed differences are due to subtle, persistent changes in autonomic function post-concussion, which manifest in altered PLR metrics, specifically regulation of pupil dilation [11]. We can only postulate the exact pathophysiology underpinning these changes; the various arms of the sympathetic and parasympathetic neural pathways of the PLR are relatively complex, and theoretically damage anywhere along its route could result in abnormal static and dynamic pupillary responsivity [15]. One possibility is that the observed changes in diameter are underpinned by damage to cranial nerves II and/or III [30]. Indeed, the PLR test assesses the neurological pathway connections and functioning of both cranial nerves II and III. In this process, light entering the eye initiates the PLR, where signals are sent to the iris sphincter muscle to regulate the amount of light reaching the retina [15,17].

Others have recorded greater end pupil diameter in adolescents with a history of concussion compared to non-concussed controls [17]. In a large case-controlled study, Master et al. [21] reported that eight out of nine PLR metrics were significantly enhanced in adolescents who were (median) 12 days post-concussion, compared to healthy controls. These authors suggest that concussion can drive changes in PLR which persist over the life span [17]. However, this study found that other metrics, including latency, constriction velocity, maximum constriction velocity, and time to 75% re-dilation, were unaffected by concussion history or biological sex. Changes to these metrics may be more apparent in the early stages post-concussion.

Adolescent females had larger initial and end pupil diameters compared to males. These metrics were most extreme in adolescent females with a history of concussion. Others have found adolescent females typically have larger maximum pupillary diameters and longer latency compared with males [17,20], with similar patterns reported in adult populations [31]. It may be that these differences are moderated further post-concussion. We know that even when exposed to a constant level of light stimulation at a consistent luminance, PLR response differs between males and females [32,33]. Responsiveness to light is regulated by an interplay of parasympathetic and sympathetic nervous systems; some previous studies have suggested that there are sex-based differences in autonomic activity (based on heart rate variability), potentially explaining the small differences we observed between males and females [34]. Other potential factors influencing pupil size include the level of retinal illumination, accommodative status, medications, and various sensorial and emotional conditions [35,36,37,38].

### Limitations

Limitations to this study include unbalanced comparison groups, particularly for sex. We recruited more males than females into the study. This could affect the generalizability of our findings to a broader population of females and should be addressed in future research. Although significant differences were found between males and females on initial and end pupillary diameters, this must be replicated in future research using a balanced representation of males and females. Pupillary assessments were performed throughout the day for logistical reasons, and although best efforts were made to ensure consistent lighting across venues, analyses did not account for diurnal variation and therefore may limit the generalisability of our findings. Participants were predominantly white, which was comparable between cohorts but limits the generalizability of our results. Due to potential variations in PLR associated with development, it is cautioned that our results should not be extended to either younger paediatric or older adult populations until a deeper understanding of the neurodevelopmental and ageing factors influencing the PLR is obtained [14,18,19]. Both younger and older age groups typically exhibit smaller pupil diameters, potentially linked to relatively lower sympathetic tone compared to adolescents. Consequently, our findings may not be applicable to these distinct populations [39].

## 5. Conclusions

A history of concussion was associated with significant differences in some PLR metrics in adolescent rugby players. Specifically, athletes with a history of concussion presented with larger end pupillary diameters compared to those with no history of concussion. This may be indicative of ongoing autonomic deficits associated with concussion. The largest differences in PLR metrics were recorded in females with a history of concussion. This highlights the importance of considering baseline differences when interpreting data in this field. Future research should seek to confirm our findings, with a focus on consistent lighting across locations, and larger sample sizes of both females with and without a history of concussion, as well as throughout the acute and subacute stages of concussion recovery.

## Figures and Tables

**Table 1:** Pupillary Light Reflex Metrics.

Initial/Max. Pupil Diameter	Steady State of the Pupil Size before the Light Stimulus
**End/Min. Pupil Diameter**	Pupil size after the maximum constriction in response to the light stimulus
**Latency**	Time to maximum constriction in response to the light stimulus
**Average Constriction Velocity**	Average speed of pupil constriction in response to the light stimulus
**Maximum Constriction Velocity**	Maximum speed of pupil constriction in response to the light stimulus
**Average Dilation Velocity**	Average speed of pupil dilation after light stimulus
**T75**	Time for pupil re-dilation from min. diameter to 75% max. diameter

**Table 2:** Pupillary Light Reflex metrics (means, SD), split by concussion history and biological sex.

PLR Metrics	No Concussion Hx		Concussion Hx		*p* Value		
	*Male (n = 59)*	*Female (n = 27)*	*Male (n = 54)*	*Female (n = 9)*	*Sex*	*Concussion History*	*Sex* * *Concussion History*
Initial Pupil Diameter (mm)	5.35 (±1.13)	5.51 (±1.26)	5.40 (±0.92)	6.22 (±1.42)	0.04 *	0.11	0.17
End Pupil Diameter (mm)	3.87 (±0.88)	3.96 (±0.91)	3.91 (±0.63)	4.64 (±1.20)	0.02 *	0.05	0.08
Latency (sec)	0.21 (±0.02)	0.21 (±0.02)	0.22 (±0.02)	0.21 (±0.02)	0.30	0.50	0.90
Average Constriction Velocity (mm/sec)	3.78 (±0.63)	3.79 (±0.54)	3.75 (±0.67)	3.92 (±0.45)	0.50	0.72	0.55
Max Constriction Velocity (mm/sec)	4.86 (±0.96)	4.87 (±0.74)	4.72 (±0.9)	4.99 (±0.71)	0.48	0.96	0.49
Average Dilation velocity (mm/sec)	1.37 (±0.24)	1.39 (±0.21)	1.32 (±0.23)	1.41 (±0.20)	0.30	0.76	0.49
T75 (sec)	1.36 (±0.56)	1.38 (±0.65)	1.36 (±0.57)	1.68 (±0.69)	0.18	0.23	0.23

*: *p* < 0.05.

## Data Availability

The data that support the findings of this study are available on request from the corresponding author. The data are not publicly available due to restrictions, e.g., they contain information that could compromise the privacy of research participants.
